# A hybrid with distributed pooling blockchain protocol for image storage

**DOI:** 10.1038/s41598-022-07494-9

**Published:** 2022-03-02

**Authors:** Feng Liu, Cheng-yi Yang, Jie Yang, De-li Kong, Ai-min Zhou, Jia-yin Qi, Zhi-bin Li

**Affiliations:** 1 School of Computer Science and Technology, East China Normal University, Shanghai, 200062 China; 2 Institute of Artificial Intelligence and Change Management, Shanghai University of International Business and Economics, Shanghai, 200336 China; 3 School of Business and Management, Shanghai International Studies University, Shanghai, 201620 China; 4 Institute of AI for Education, East China Normal University, Shanghai, 200062 China

**Keywords:** Computer science, Electrical and electronic engineering

## Abstract

As a distributed storage scheme, the blockchain network lacks storage space has been a long-term concern in this field. At present, there are relatively few research on algorithms and protocols to reduce the storage requirement of blockchain, and the existing research has limitations such as sacrificing fault tolerance performance and raising time cost, which need to be further improved. Facing the above problems, this paper proposes a protocol based on Distributed Image Storage Protocol (DISP), which can effectively improve blockchain storage space and reduces computational costs in the help of InterPlanetary File System (IPFS). In order to prove the feasibility of the protocol, we make full use of IPFS and distributed database to design a simulation experiment for blockchain. Through distributed pooling (DP) algorithm in this protocol, we can divide image evidence into recognizable several small files and stored in several nodes. And these files can be restored to lossless original documents again by inverse distributed pooling (IDP) algorithm after authorization. These advantages in performance create conditions for large scale industrial and commercial applications.

## Introduction

Blockchain technology is constantly from the theoretical exploration to application landing, and a large number of decentralized applications (DAPP) have emerged in this stage. With the emergence of Turing-complete programming language in blockchain, such as smart contracts, the demand for storage space has increased dramatically while the function is more abundant^1^. In the process of blockchain application diversification, the problem of insufficient storage space is increasingly prominent. At present, the data storage capacity of Ethereum has reached 540 GB, and shows the trend of accelerated growth. How to make full use of the limited distributed storage space has become an urgent problem.

In a traditional centralized storage scheme, data storage is often transferred through the client to a centralized server for storage. Once the server is attacked, then the user will face huge losses. Compared with centralized storage, the advantages of distributed storage scheme are that the risk of privacy leakage is reduced; data storage is sustainable, the storage cost is allocated to the whole network; and the central enterprise does not need to invest a huge amount of cost to maintain storage facilities. Due to the shortage of distributed storage space, users need to pay for the uploaded and downloaded files, which makes the cost of using blockchain to transfer files much higher than that of internet centralized server. In the long term, this limits the popularity of blockchain technology in the current society.

What’s more, in the light of the present research situation, most of the schemes to reduce the space requirement are based on compressed sensing technology, but nevertheless the compression method of this kind of method is still lossy in the vast majority of cases. The files restored after compression are different from the original files, and the progress of compressed sensing technology only reduces these differences as much as possible. In blockchain, which is a system used to store account transaction records, electronic contracts, invoice documents, security and other important information, is faced with a greater risk of losing key information. Meanwhile, compressed sensing needs to spend a lot of time to make up for the damaged part of the file which is difficult to meet the needs of industrial applications in terms of efficiency. Dai et al.^2^ proposed a distributed storage framework based on random linear network coding (RLNC) in blockchain. In this method, the storage files in a block are divided into smaller blocks and stored in multiple nodes by random linear network coding (RLNC), which reduces the storage space requirement of a single node.

However, the RLNC method is just a preliminary distributed storage framework, which lacks the consideration of the performance on complete restoration. Moreover, we notice that the scheme does not consider the problem of reducing the fault tolerance performance caused by the decentralized storage of single file. To be specific, as long as there is no response, error or attack on the data on a node, the user can no longer clearly know the content of the file, and the blockchain storage will lose its function as a proof of storage.

In order to solve the above problems, we propose a distributed storage protocol for blockchain based on the pooling algorithm and its inverse process. The advantages of the improvement are as follows:

- It effectively solves the problem of insufficient storage space and reduces the cost of storage on the blockchain with the help of IPFS. By accessing the IPFS network, the data on the blockchain will become smaller^3^, thus the storage space required by a single node will also be reduced.
- It greatly improves fault tolerance. If only one piece of data is simply divided into multiple parts in order, as long as the data stored in one node is lost, the whole data can no longer be obtained. The data stored in any node can be identified.
- It can be restored completely and losslessly. Each node does not lose data, even if some information is lost, it can be restored according to the proportion of information retained.

## Related work

The traditional data compression method is mainly based on the compressed sensing theory^4^, a lossy compression method for sparse signals^5^. That is to find the sparse representation of the signal lower than Nyquist sampling rate, and restore the signal as undistorted as possible by reconstruction. Eldar and Kutyniok^6^ list some signal reconstruction algorithms for solving local optimum by greedy pursuit, including matching pursuit (MP)^7^, orthogonal matching pursuit (OMP)^8^ and subspace pursuit (SP)^9^. OMP algorithm has the most far-reaching influence on the future research in the field of compressed sensing, which derived a variety of improved versions of the algorithm. For example, generalized orthogonal matching pursuit (GOMP)^10^ is formed by enlarging the range of residual selection in iteration; regularized orthogonal matching pursuit (ROMP)^11^ is formed by replacing single column iteration with multi-column iteration; stage orthogonal matching pursuit (StOMP)^12^ is formed by replacing single atom iteration with multiatom iteration; compressed sample matching pursuit (CoSaMP)^13^ is formed by setting the atoms selected in each iteration will be discarded instead of being reserved in the next iteration. There are also some dictionary learning algorithms^14^, such as principal component analysis (PCA), Ksingular value decomposition (KSVD)^15^, optimal direction method, greedy adaptive dictionary (GAD)^16^, etc.

Although compressed sensing technology can achieve similar effect to the original file through various restoration algorithms, the limitations of this method are obvious. First of all, the compression condition must be sparse signal, such as images with most gray value of 0 or audio signals with most amplitude value of 0. Secondly, any restoration algorithm needs to reconstruct each row and column through the estimation technology, which takes a long time and is hard to meet the needs of large-scale industrial applications. Finally, considering the requirement of restoration quality, the compression ratio of compressed sensing technology is still limited. For the blockchain network with geometric growth of data volume, how to meet the demand of releasing storage space reasonably is very difficult.

With the development of blockchain, the data stored on chain is usually some important supporting documents, such as business contracts, legal documents, voice evidence, etc. In order to ensure the storage security and lossless restoration of files, the demand for reducing the storage cost, transmission cost and node space by transferring storage gradually appears.

Jafari and Plumbley^17^ proposed a blockchain data compression scheme for IPFS, which enables most transactions to be confirmed locally. Only a small number of transactions need to access the IPFS network, and the compression ratio in bitcoin network reaches 0.0817. This also provides a feasible idea for us to prove that the protocol can improve the blockchain storage space and storage cost with the help of IPFS. In 2018^18^, a distributed cooperative spectrum sensing scheme is proposed, and one hop information fusion is realized by using spatial information fusion technology based on average hops and distributed computing technology. Then, the fusion supporting estimation results are used as prior information to guide the next local signal reconstruction, and the algorithm is implemented by our weighted binary iterative hard threshold (BIHT) algorithm. Local signal reconstruction and distributed fusion of supporting information alternate until reliable spectrum detection is achieved. Yan et al.^19^ also compressed the bitcoin transaction records by replacing the hash pointer of the previous block hash value with the index pointer, which can reduce the storage space by 12.71%. On the basis of synchronous OMP (SOMP) algorithm and KSVD dictionary learning, an improved version of adaptive joint reconstruction algorithm DCS SOMP was proposed^20^. SOMP is used to estimate and reconstruct the sampled data, and KSVD method can update the over complete dictionary many times and reduce the error between the reconstructed signal and the original signal.

Although the above-mentioned methods have focused on solving the storage problem in blockchain or distributed network, there are still some problems to be further studied, such as the time cost of algorithms, the fault tolerant performance of storage nodes, and the redundant of information^21^. Therefore, this paper proposes a solution that can reduce the storage space requirement of blockchain nodes with low time overhead, high fault tolerance and lossless restoration, so as to help the wider industrial and commercial applications of blockchain network.

### Distributed image storage protocol based on blockchain

**Figure 1:**
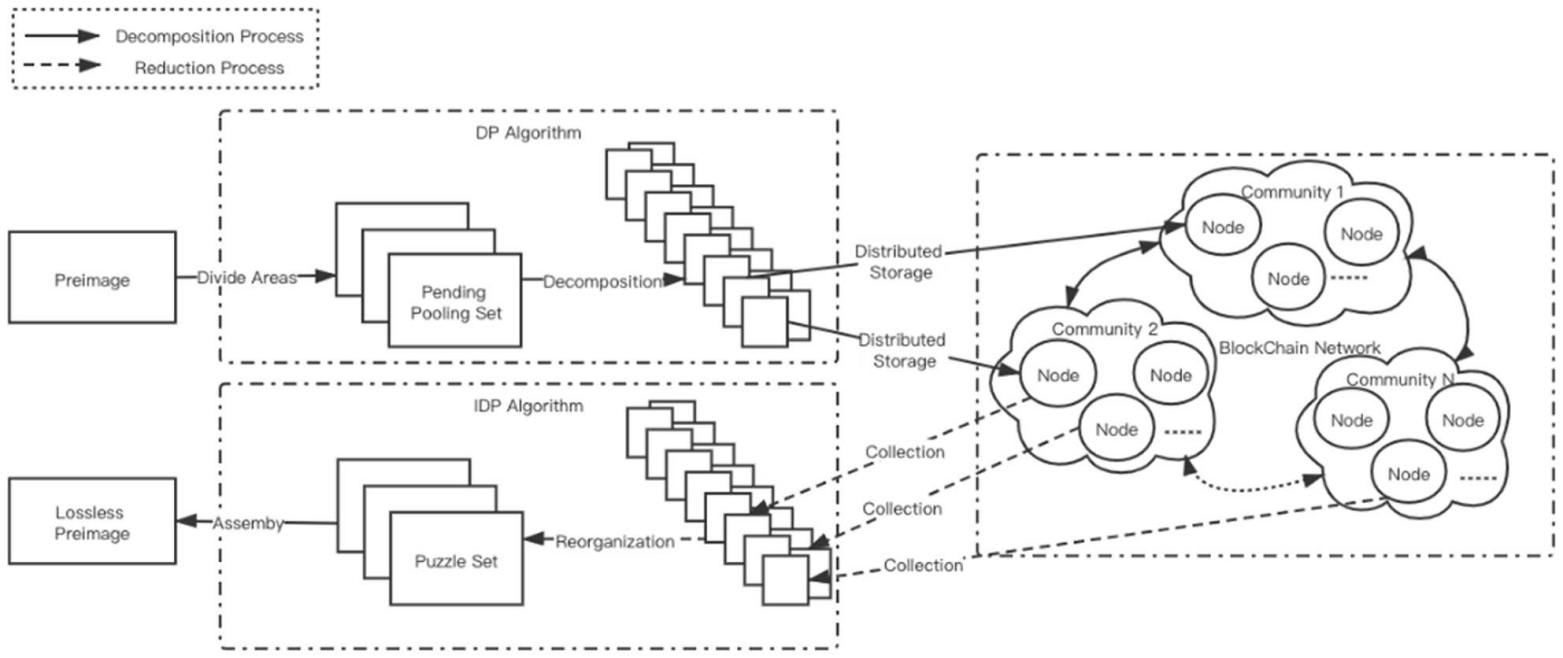
Architecture of distributed image storage protocol.

In this section, we will introduce the architecture of the distributed image storage protocol based on blockchain. Through the architecture overview, the general process of compressing and storing images by this protocol will be presented, as shown in Fig. 1. What’s more, the community information on blockchain will also be analyzed in detail.

#### Architecture overview

This protocol is designed optional and not mandatory, and users who do not accept DISP protocol can still use the traditional way of full redundancy storage. However, users who sign the agreement can enjoy the benefits of space saving without reducing safety performance. In the traditional blockchain network each user must store exactly the same data to ensure the fault tolerance of the whole network and avoid the bifurcation caused by malicious attack or fraud. DISP protocol changes the full redundancy storage relative to individuals into the community level full redundancy, namely there is no redundancy for the data stored in each node of one community.

In DISP, distributed storage ensures that all data will not be lost when a few nodes are attacked or fail so that the performance of data security can be enhanced. And the distributed pooling algorithm reduces the data redundancy of distributed storage and saves storage space greatly.

#### Community formation

In the preprocessing stage before the implementation of distributed pooling algorithm, the original image is first divided into several pooling regions according to the shape of the pooling kernel to form a set of pooling regions to be processed. Then the image can be divided into several parts and stored in several nodes through distributed pooling algorithm.

The address who signs the protocol will be gathered to form different communities and the number of nodes in each community is determined by the number of pooled images obtained after the decomposition algorithm. Under a certain compression ratio, every piece of data is still recognizable as well as can be restored losslessly by compressed sensing or super resolution representation. If all the pieces of data in the community is collected, then the original data can be restored losslessly by the inverse operation of DP algorithm.

The data saved by each node is different from that of other nodes in the community, otherwise, the node will be divided into another community. Each node in a community can see the whole picture of the data. If the corresponding pieces of data stored by each node in the community are collected together, then the origin data can be restored losslessly after invoking evidence phase.

### Pooling algorithm for image

In this section, we will try to design a compression algorithm that can restore losslessly in the future, which is called distributed pooling. The idea comes from a down-sampling algorithm called pooling, but distributed pooling algorithm is essentially different from any existing pooling algorithm. An image will be split into several pictures and stored in different addresses of the distributed network, which enables each node to pay less storage cost and the original image can be synthesized losslessly if necessary.

#### Pooling algorithm

The early pooling algorithm simulates the characteristics of receptive field of cortical neurons in primary visual area, and extracts features from original images using the principle of independent maximization of sparse coding^22,23^. With the rise of data driven methods, related research began to focus on the nonlinear characteristics of optimal pooling and probabilistic representation of subspace size. By maximizing the independence between projection norms on a linear subspace, Reference^24^ explained the emergence of spatial phase shift in variance, and used independent component analysis (ICA) modeling:

1 $$\begin{array}{*{20}c} {I \left( {x,y} \right) = \mathop \sum \limits_{i = 1}^{m} b_{i} \left( {x,y} \right)s_{i} } \\ \end{array}$$

where $s_{i}$ is the randomly generated component coefficient, $b_{i} \left( {x,y} \right)$ is the base equation representing the feature mapping. Then a single channel gray image can be expressed as a linear combination of multiple characteristic components. $s_{i}$ can be expressed as a filter to extract features from the image, namely the inner product of filter coefficients and region pixels:

2 $$\begin{array}{*{20}c} {s_{i} = \mathop \sum \limits_{x,y} w_{i} \left( {x,y} \right)I\left( {x,y} \right) = \left\langle {w_{i} ,I} \right\rangle } \\ \end{array}$$

#### Lp norm pooling

With the further research on representation learning and signal reduction, the introduction of Lp norm increases the interpretability of pooling definition. The n dimensional spherical form formed by the contour lines of Lp norm in Euclidean space is described as Lp ellipsoidal distribution^25^. This concept was introduced into Lp norm subspace analysis (LpISA), which has formed a mathematical expression of pooling:

3 $$\begin{array}{*{20}c} {\left( {\left| {x_{{I_{i} }} } \right|^{p} + \cdots + \left| {x_{{I_{l} }} } \right|^{p} } \right)^{\frac{1}{p}} } \\ \end{array}$$

When $p = 1$, the Lp norm corresponds to the average pooling. And when $p \to \infty$, Lp norm is the form of maximum pooling, which can be expressed as:

4 $$\begin{array}{*{20}c} {max\left( {\left| {x_{{I_{i} }} \left| {, \cdots ,} \right|x_{{I_{l} }} } \right|} \right)} \\ \end{array}$$

Estrach et al.^26^ conducted an experiment on the MINIST image data set based on the L1, L2 and Lp norm respectively. By comparing the reversibility after pooling, he concluded that the difficulty of recovery of the three methods was roughly the same, and proposed a more general pooling operator based on $K$ disjoint pixel block, where $s$ is the stride, which is similar to convolution operation, and $K$ is the size of the convolution kernel:

5 $$\begin{array}{*{20}c} {I\left( {i,j} \right) = \left[ {\mathop \sum \limits_{x = 1}^{K} \mathop \sum \limits_{y = 1}^{K} I \left( {s \cdot i + x,s \cdot j + y} \right)^{p} } \right]^{\frac{1}{p}} } \\ \end{array}$$

#### Distributed pooling

**Figure 2:**
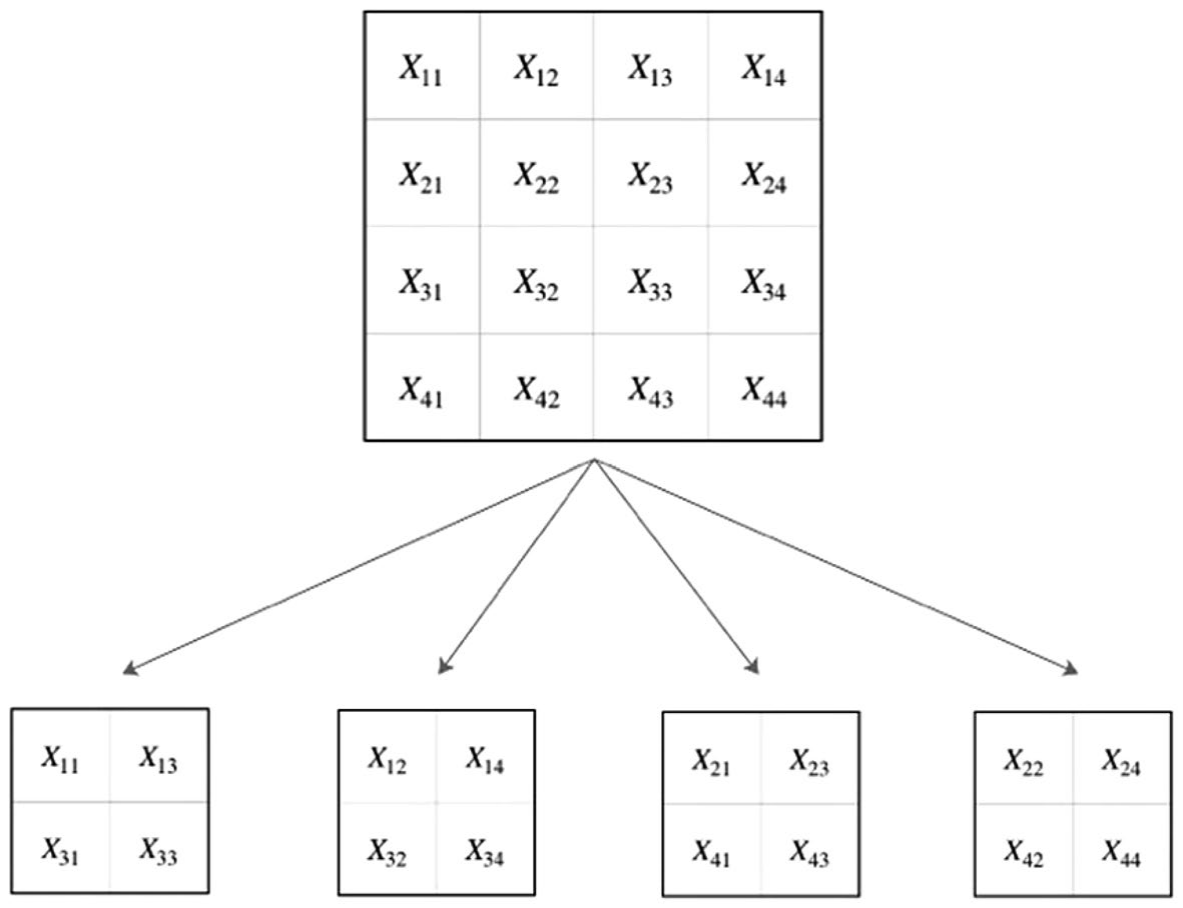
Sketch map of distributed pooling algorithm.

The proposed method named is distributed pooling because its idea comes from pooling algorithm, which is essentially kind of like an image decomposition algorithm. As shown in Fig. 2, an origin picture will be divided into several ordered regions, and then the pixels of the same position in each region will be extracted to recompose a new smaller picture. This process can be repeated many times until all the pixels in the original image has been extracted. The number of decomposed images is equal to the number of pixels contained in the ordered region.

Such an operation process is a pooling algorithm for one specific decomposed image, but this process is a pixel level decomposition for all decomposed images. In addition, the decomposed images can be completely restored to lossless images, which is impossible by traditional pooling algorithm for they will discard pixels.

Let $X$ be an image to be decomposed for distributed storage. $f_{s.t}$ are small square areas demarcated on the original image $X$, where $s$ and $t$ represent the division area of row $s$ and column $t$ on the original image. Each pixel in the region $f_{s.t}$ can be denoted as $f_{s.t,u,v}$. where $u$ and $v$ represent the row position and column position of the pixel in region $f_{s.t}$. The size of $f_{s,t}$, namely $max\left\{ u \right\}{ } \times { }max\left\{ v \right\}$, is called pooling kernel size, which determines the size of the decomposed image.

Let $G_{k,l}$ be the decomposed image, where $k$ and $l$ mean which pixel is from in the division area $f_{s.t}$. Each pixel in $G_{k,l}$ can be denoted as $G_{k,l,i,j}$, where the subscripts $i$,${ }j$ represents the row position and the column position of the pixels in decomposed image $G_{k,l}$ . $N$ is the number of images after decomposition, which satisfies $N{ } = { }max\left\{ k \right\} \times { }max\left\{ l \right\}$. The whole pooling process follows the following expression:

6 $$\begin{array}{*{20}c} {G_{k,l,i,j} = f_{i,j,k,l} } \\ \end{array}$$

Therefore, $G_{1,1,1,2} = { }f_{1,2,1,1}$ means that the pixel in first row and second column in the first decomposed image $G_{1,1}$ is derive from the first row and first column in division area $f_{1,2}$. Equation (6) describes the operation rules of Fig. 1 in the form of mathematical pixel expression.

Different from other Lp norm pooling, the distributed pooling algorithm is a method to extract pixels orderly at the same position in each pooling area from original tensors, which can also be understood as a down-sampling method according to certain rules. Different from the common pooling algorithm, such a down-sampling algorithm will carry out multiple times according to the different positions of each pooling area until all the pixel positions on each pooling region have been extracted. Each image after pooling is different, but they are all down-sampling representations of the original image.

#### Main implementation steps

**Figure 3:**
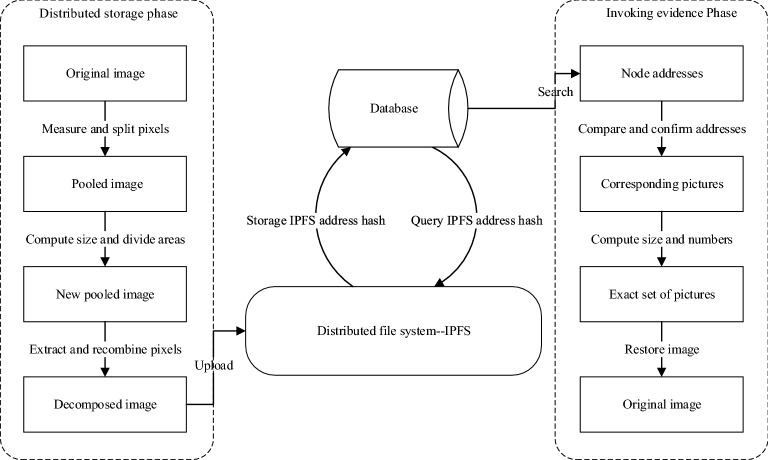
Address writing and calling through IPFS.

When building a decentralized application (DAPP), the widely adopted approach is to store the hash values on the blockchain and store the information that needs to be stored in the centralized database. In this way, storage becomes a big shortcoming in decentralized applications and is a vulnerable link in the blockchain network. Fortunately, IPFS presents a solution: one can use it to store files and place a unique and permanently available IPFS address into a blockchain transaction. By this means, it is not necessary to place the space-hungry data itself on the blockchain. On the other hand, IPFS can also assist various different blockchain networks in transferring information and files which can improve the scalability of blockchain. Based on these advantages about IPFS, we design the main contents of distributed pooling include Distributed Storage Phase and Invoking Evidence Phase, as shown in Fig. 3.

The left part of Fig. 3 is Distributed Storage Phase, and the right part of Fig. 3 is Invoking Evidence Phase which is used to recover the original image by inverse distributed pooling operation. After finishing the distribute storage phase, querying the corresponding image hash addresses will be easier from database. Through the distributed characteristics of IPFS, the hash value of addresses is unique and trusted. Thus, in the condition of trusted nodes, we can make use of inverse distributed pooling operation to recover the original image after confirming the correction of node addresses.

## Results and discussion

Traditional blockchain storage method adopts full redundancy storage in order to ensure its security. Namely, each node in the blockchain network is required to store the same electronic certificate or transaction information so as to realize the distributed authentication and decentralized transaction of the blockchain community. In many consensus mechanisms such as Proof of Work (PoW), once the information stored by nodes is inconsistent, it will cause fork. Although this way ensures the security and non-repudiation of the blockchain, along with the in-creasing number of blockchain users and the diversified implementation of de-centralized applications, this kind of high redundancy method is not sustainable in the face of the growing demand for file transmission of data records.

The implementation of DISP storage protocol greatly reduces the storage space requirement of single node in distributed network. We note that the distributed storage scenario is quite suitable for blockchain, which is not acceptable in a centralized storage scenario. The product of the space occupied by the distributed several files and the total storage space occupied is actually larger than the original data, so this method is meaningless in the centralized storage environment. However, in the fully redundant blockchain scenario, the nodes in the network no longer need to store all the files, but only need to store the identifiable part of DP algorithm, which greatly reduces the space occupation. Even if a few nodes suffer from malicious attack and cannot be used for recovery of the original image, they can still be reorganized with the help of IDP algorithm in this protocol.

The output after pixel level reorganization by DISP algorithm is completely free of information. The performance of the down-sampling and restoration technology of DISP algorithm is better than the compressed sensing technology based on convex optimization method in terms of computational cost and time cost. DISP algorithm can instantly complete the orderly decomposition and lossless restoration of documents under CPU operation, which provides conditions for more extensive industrial applications.

### Experiment

In order to verify the progress of DISP in saving storage space and improving fault tolerance performance, we designed an experiment about contrast transmission based on IPFS protocol. In the same hardware environment, file transmission is realized by DISP protocol. The size of pooling kernel, time cost, change of storage room and performance of fault tolerance are recorded in Table 1. The relevant experimental codes can be referred in the footnotes area of this page, and the hardware environment of configuration used in the experiment is as follows:

- Memory: 32 GB 2400 MHz DDR4
- CPU: 8core Intel Core i9 2.3 GHz
- Hard disk: 1 TB
- System environment: MacOS Catalina 10.15.61

**Table 1:** Comparison of DP effects under different pooling kernel parameters.

Kernel size	Time consuming	Storage size (Kb)	Fault tolerance	Quantity
2 × 2	0.1475	12.86	recognizable	4
4 × 4	0.1655	4.89	recognizable	16
8 × 8	0.1587	2.05	blurring	64
16 × 16	0.1547	1.06	unrecognizable	256
2 × 4	0.1537	8.05	recognizable	8
4 × 2	0.1534	7.67	recognizable	8
4 × 8	0.1544	3.14	blurring	32
8 × 4	0.1530	3.02	blurring	32
2 × 8	0.1573	5.01	barely discerning	16
8 × 2	0.1502	4.70	barely discerning	16

The original data used in the experiment is a color image called Lena with 512 × 512 pixels, which is known as the benchmark image in document compression. In order to make the test more stable, the three channels called RGB are combined into a single channel format of JPG as gray scale image and storage space occupied is 36.32 KB.

**Figure 4:**
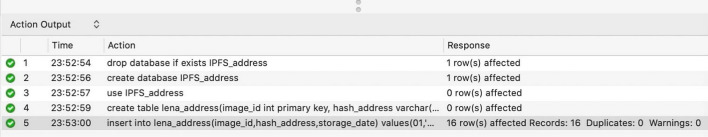
The result feedback of adding address into database.

In the stored procedure, the decomposed images obtained by distributed pooling need to be stored in different nodes for distributed storge. Therefore, we introduced a distributed file system named Interplanetary File System (IPFS) which can store and share data in blockchain network. To uniquely identify each file, IPFS give permission to use content-addressing in distributed web. It is easily to get the address in hash form by calling the IPFS command instantaneously. The address returned from IPFS will be recorded in one table in the database and the addresses of pooled images recorded in each table are obtained from the decomposition of the same original image, which is for the convenience of searching all the small images when restoring losslessly. The result of succeed storing is shown in Fig. 4.

**Figure 5:**
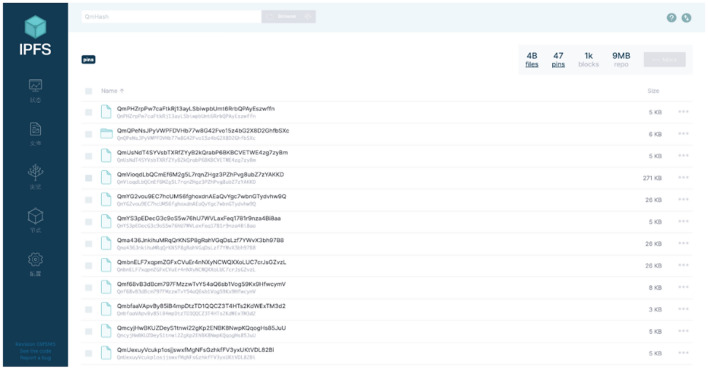
Test results on IPFS.

After the operation of DP, N images with size of $\frac{W}{\sqrt N } \times \frac{H}{\sqrt N }$ are formed and stored in N nodes. The original image can be restored losslessly by collecting the information stored in these nodes and implement interpolation, which is the algorithm of IDP. In order to show the effect of different pooling kernel on the experimental results, the pooling kernel size of $2 \times 2$,${ }4{ } \times { }4$, $8{ } \times { }8$ was selected to conduct the experiment with DP algorithm, and the test results on IPFS are shown in the Fig. 5.

**Figure 6:**
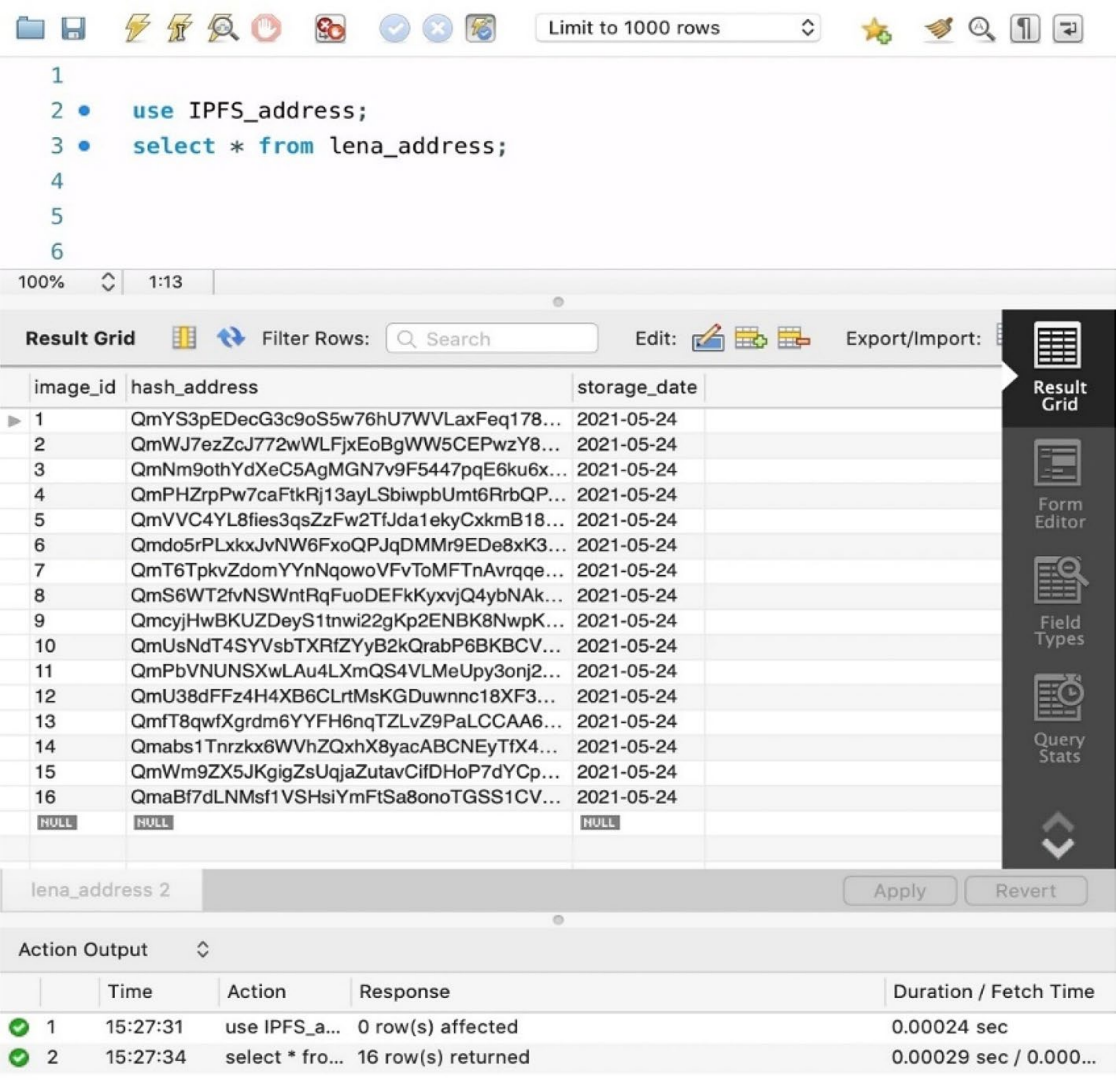
Query IPFS address from database.

In the process of query, the image address stored in IPFS will be returned after inputting the query instruction in workbench which is a simulation environment of database operation instruction. Once the query results are obtained like Fig. 6, all the decomposed images can be downloaded through IPFS instructions as shown in Fig. 4.

In order to provide more options for use, this protocol additionally provides a special function called erasable. Although we can’t delete the files stored on the blockchain, the storage address of the decomposed image is the hash in the database. This means that we can’t find the decomposed image by deleting the hash address stored in the database. The deletion effect on the block can be realized in this way, although this approach is not really deleted and essentially a way of data isolation.

DISP security protocol decomposes an image into several parts and stored in several nodes, and the number of divided parts is determined by the width and height of the pooling kernel. Table 1 shows the experimental results of each parts obtained in algorithm of DP under different pooling kernel parameters. The results in the table show that the storage space occupied by each image after allocating decreases with the increase of pooling kernel, but the resolution corresponding to the image will be reduced accordingly. When the size of pooling kernel increases to $8 \times 8$, it can be observed that the image is still visible even though the size of image is smaller. While the height or width of pooling kernel increases to 16, the benchmark image with $512 \times 512$ pixels will be decomposed into 256 images with size of $32 \times 32$ , and the contents on the image will not be recognized by the naked eye.

In order to show the good performance of DISP protocol, we also conduct the contrast experiment with com pressed sample matching pursuit (CoSaMP)^13^, subspace tracking (SP)^9^, threshold iterative method (IHT)^26^ and iterative reweighted least squares (IRLS)^27^ algorithm at sparse ratio of 6.25% and 25%. These algorithms first sparsely sample the image information, and then reconstruct each column of the image in the form of optimization algorithm. The experimental results can be shown in Table 2, time consuming and reconstruction accuracy is recorded as the performance index of these algorithms. By calculating the reconstruction loss, we find that the DISP achieved accuracy of 100%, which shows that this protocol can truly realize lossless restoration of the original image. The detail figures will be shown in the supplementary file.

**Table 2:** Performance of different methods.

Methods	Sparse ratio (%)	Type	Time consuming (s)	Reconstruction accuracy (%)
CoSaMP	6.25	Compressed sensing	3.85 ± 0.04	3.64
IHT	6.25	Compressed sensing	0.20 ± 0.01	10.83
IRLS	6.25	Compressed sensing	2.41 ± 0.03	62.07
SP	6.25	Compressed sensing	0.56 ± 0.02	71.91
DISP	6.25	Distributed down-sampling	0.24 ± 0.01	100
CoSaMP	25	Compressed sensing	54.50 ± 0.5	19.41
IHT	25	Compressed sensing	0.96 ± 0.02	55.38
IRLS	25	Compressed sensing	23.89 ± 0.1	85.92
SP	25	Compressed sensing	3.96 ± 0.04	86.45
DISP	25	Distributed down-sampling	0.25 ± 0.01	100

## Conclusion

In this paper, we design a low storage requirement protocol based on blockchain named DISP, which contains DP algorithm and its inverse operation IDP algorithm capable of adapting to distributed scenarios. DP decomposed a high pixel image into several low pixel images by selecting pixels in order in each pooling area, and the generated images saved by each node are still recognizable. IDP reorganizes the distributed information saved by each node according to the original order so that it can realize the lossless restoration of the original documents.

The next work will focus on the automatic selection of super parameters such as the optimal size of pooling kernel and the corresponding fault tolerance performance evaluation, so as to enhance the value of DISP protocol in practical application and make it more attractive to users. Meanwhile, this protocol does not give much consideration to the aspect of security and privacy of single node. The next will incorporate some cryptographic techniques to prevent a large number of nodes from losing and leaking the original image information, so as to enhance the security of the protocol itself finally. In addition, the protocol does not attempt to test and analyze larger data information such as audio and video. In order to meet the ever-changing business needs, authors will also try our best to improve the protocol so that it can compress and store audio and video.

## Supplementary Information

Supplementary Information.
