# Author Correction: A hybrid with distributed pooling blockchain protocol for image storage

**DOI:** 10.1038/s41598-022-09492-3

**Published:** 2022-03-28

**Authors:** Feng Liu, Cheng-yi Yang, Jie Yang, De-li Kong, Ai-min Zhou, Jia-yin Qi, Zhi-bin Li

**Affiliations:** 1 School of Computer Science and Technology, East China Normal University, Shanghai, 200062 China; 2 Institute of Artificial Intelligence and Change Management, Shanghai University of International Business and Economics, Shanghai, 200336 China; 3 School of Business and Management, Shanghai International Studies University, Shanghai, 201620 China; 4 Institute of AI for Education, East China Normal University, Shanghai, 200062 China

Correction to: *Scientific Reports* 10.1038/s41598-022-07494-9 published online 02 March 2022

The original version of this Article contained an error in the spelling of the author Feng Liu which was incorrectly given as Liu Feng.

The original Article has been corrected.

