# Supplementary material for: A hybrid with distributed pooling blockchain protocol for image storage
Source: Sci Rep. 2022 Mar 2;12:3457. doi: 10.1038/s41598-022-07494-9 (PMC8891285; doi:10.1038/s41598-022-07494-9)
Supplement: Supplementary file 1 — Supplementary Information. [file 41598_2022_7494_MOESM1_ESM.pdf]

Experimental results of compressed sensing method

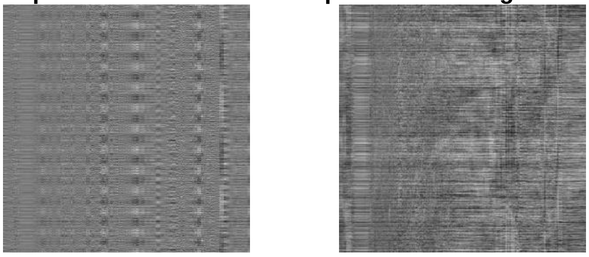

Supplementary Figure S1: Reconstruction with sparsity ratio of 6.25% and 25% by CoSaMP

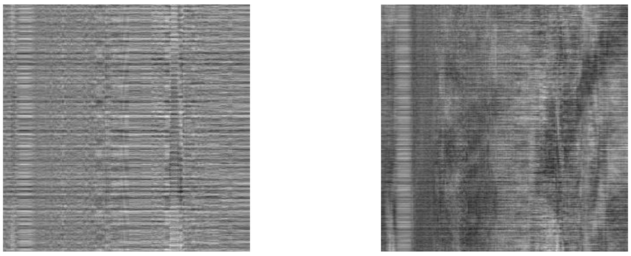

Supplementary Figure S2: Reconstruction with sparsity ratio of 6.25% and 25% by IHT

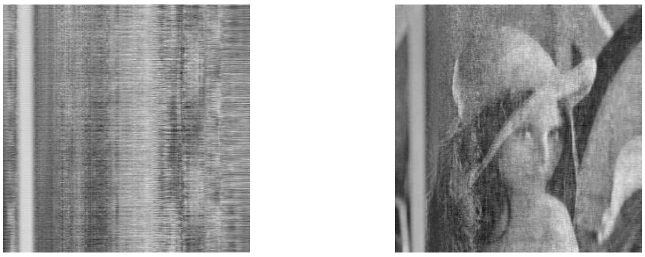

Supplementary Figure S3: Reconstruction with sparsity ratio of 6.25% and 25% by IRLS

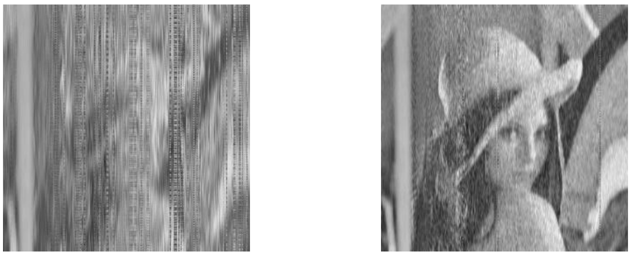

Supplementary Figure S4: Reconstruction with sparsity ratio of 6.25% and 25% by SP
